# Editorial: Recent advances in papillary thyroid carcinoma: Progression, treatment and survival predictors

**DOI:** 10.3389/fendo.2023.1163309

**Published:** 2023-03-07

**Authors:** Erivelto Martinho Volpi, Margarita Carmen Ramirez-Ortega, Jose Federico Carrillo

**Affiliations:** ^1^ Department of Head and Neck, Oswaldo Cruz German Hospital, São Paulo, Brazil; ^2^ Centro De Referencia No Ensino Do Diagnóstico Por Imagem (CETRUS), São Paulo, Brazil; ^3^ Research Division, Department of Pharmacology, Instituto Nacional de Cardiologia Ignacio Chavez, Ciudad de México, Mexico; ^4^ Department of Head and Neck, National Institute of Cancerology (INCAN), Mexico City, Mexico

**Keywords:** differentiated thyroid carcinoma, transcriptomics in papillary thyroid carcinoma, ultrasound nomogram in thyroid carcinoma, aggressive histology variants in thyroid carcinoma, central neck dissection in papillary thyroid carcinoma, BRAFV600E mutation in papillary thyroid carcinoma

In this Research Topic from Frontiers in Endocrinology on *Recent Advances in Papillary Thyroid Carcinoma: Progression, Treatment and Survival Predictors*, several reports are gathered with a common aim, which is the etiopathogenic mechanisms that trigger the development and progression of this malignancy.

Some of them like the body mass index (BMI) presented by Economides et al. have been unfrequently explored. The meta-analyses presented constitutes a pioneering effort to explore the mechanisms of development through an understanding of its link with prognostic factors. These were the tumor size, extrathyroid invasion, the T N M classification, and multifocality, which, with an intermediate value in terms of odds ratios, demonstrate the relevance of a higher BMI and the possible relation already reported with the female gender, where thyroid carcinoma has greater incidence. Additionally, the potential mitogenic effect of estrogens in thyroid cancer, which has been already reported (1), is considered, and its increased frequency in patients with breast cancer was explained in the presence of a hyperestrogenic state.

The nature of different subpopulations of cells and its relation to malignant follicular cells is described in the report by Yan et al., showing its dynamics and heterogeneity, which explains its particular behavior in primary and metastatic lesions: indolent—because of the intense recruitment of interstitial cells by cancer cells in primary and metastatic sites—and aggressive when myofibroblastic cancer–associated fibroblasts increase in same areas and could help to develop different medical strategies to treat these malignancies utilizing the transcriptomic analyses of the specimen. The potential role of associated T and B lymphocytes in metastatic and primary malignant tissues is described as well, and the implications for new drug inclusion in medical therapies is explained.

A controversial issue exists regarding the male gender as a high-risk prognostic factor in differentiated thyroid cancer patients. While some reports (2) support its role, others disagree (3). An interesting study by Siraj et al. describes a cohort of patients with a relatively young age, Middle Eastern nationality, and median follow-up longer than other reports (9.5 years), where they find in multivariate analyses a significant association of shorter recurrence free survival (RFS) with the male sex. This underlines the role of this factor, which, in recent articles, is reported more frequently associated with a higher risk of recurrence, especially in Eastern countries (2).

Pioneering efforts are currently in process to discern the role of gene mutations in thyroid cancer development and, specifically, its association to high-risk factors, including histopathology. The manuscript from Poma et al. describes the hobnail papillary thyroid cancer variant in this regard. They demonstrate the association of this histology to high-risk factors as well as the presence of the telomerase reverse transcriptase (TERT) promoter mutation, which confirms recent reports that ascribe a higher significance to this mutation in contrast to the v-raf murine sarcoma viral oncogene homolog B1, valine to a glutamic acid substitution at position 600 mutation (BRAF V-600 mutation), whose prognostic impact at present is controversial (4, 5).

Although restricted to patients with suspicious thyroid nodules, before treatment was performed, the study by Ni et al. attempts to describe a prognostic score for malignancy according to the ultrasonographic characteristics of possibly metastatic lymph nodes, which included a long vs. short axis ≤2, hyperechogenicity, cystic changes, calcifications, an absent hilum, and abnormal flow. Some of these sonographic alterations have already been described by others (6). However, the statistical assessment (including the receiver operating characteristic (ROC) curve associated) and the risk groups described add to the planning of surgical strategy in the initial management of thyroid nodules and cancer. Furthermore, their findings could be extrapolated to discern which thyroid carcinoma patients could be amenable to a fine needle aspiration biopsy in case of the suspicions of the recurrence of lymph nodes either in the central or lateral compartments, which could lead to its eventual resection.

In an interesting report by Li et al., a cohort of intermediate-risk thyroid cancer patients who received complete resective surgery and 131 Iodine (I131) therapy is classified as excellent response (ER) and non-excellent response (NER) cases, according to thyroglobulin levels and image studies. They demonstrate that the primary tumor size, metastatic lymph node number, lymph node ratio, lymph node location, and stimulated thyroglobulin levels (ps-Tg) above 10 ng/ml are significant factors predictive of ER. Another interesting finding in this report is that the presence of the BRAF-v600 mutation was not associated to an NER, which adds relevance to recent analyses that underline the lack of significance of this mutation regarding its association to high-risk factors and a decreased recurrence rate and survival.

Wang et al. present meta-analyses on the efficiency and complications of prophylactic central neck dissection (PCND) for papillary thyroid carcinoma (PTC). Although including patients with small tumors and with no clear-cut differentiation of these from T3–T4 cases—a hindrance detected in many studies and pointed out by others (7)—they find a lower recurrence rate in cases who had total thyroidectomy (TT) and PCND than in those cases subjected to TT with no PCND, as well as a low complication rate, specifically regarding recurrent nerve damage, with no difference when compared with PCND cases. Of note, transient hypoparathyroidism was more frequent in the group that received TT plus PCND but permanent hypoparathyroidism presented equally, a fact that has been reported more frequently in recent studies (8). All of these facts warrant prospective controlled studies on this subject (9, 10).

In summary, new factors, like patients’ gender, metastatic lymph nodes, and body mass index, which in the past were described as non-significant, take new perspective and relevance in the management of PTC. Transcriptomic analyses and different cell lines’ equilibrium are described, as well as the real value of some gene mutations like BRAF V-600, which appears, according to recent reports, of indeterminate relevance. Moreover, the role of tumor markers—like thyroglobulin and antithyroglobulin antibodies—as a method of the quality of response to treatment and its potential for early recurrence detection are emphasized. New ultrasound refinements are described, which have the potential to improve early detection and participate in the treatment of regional recurrences, like radiofrequency ablation and the wire-guided resection of metastatic recurrent lymph nodes.

## Author contributions

JC: Design, conceptualization, editing, final format of this manuscript. EV: Proposal of Topic, Design, conceptualization, editing and final format of this manuscript. MR-O: Editing, final format of manuscript, conceptualization of basic science in this manuscript. All authors contributed to the article and approved the submitted version
